# Multi-modal management of dental fluorosis in a family: A case series

**DOI:** 10.1097/MD.0000000000042082

**Published:** 2025-04-11

**Authors:** Ghadeer Saleh Alwadai, Salha Hussein Huaylah, Mashael Khalid Althobati, Fahad Amer AlMutairi

**Affiliations:** a Department of Restorative Dental Science, College of Dentistry, King Khalid University, Abha, Saudi Arabia; b Department of Restorative Dentistry, Ministry of Health, Abha, Saudi Arabia; c Department of Prosthodontic Dentistry, Ministry of Health, Abha, Saudi Arabia; d Department of Family Dentistry, Ministry of Health, Abha, Saudi Arabia.

**Keywords:** bleaching, dental fluorosis, dental treatment, laminate veneers

## Abstract

**Rationale:**

This case series addresses the management of dental fluorosis, a condition caused by excessive fluoride intake during tooth development. The 3 patients in this report, all from the same family, exhibited varying degrees of dental fluorosis, impacting both aesthetics and function. Treatments ranged from conservative approaches like bleaching and macro-abrasion to more extensive restorative options like veneers and crowns, aiming to improve dental appearance and oral health.

**Patient concerns:**

All 3 patients were concerned with the discoloration and aesthetic issues caused by dental fluorosis. Each expressed a desire to improve their smile and address any functional impacts on their teeth.

**Diagnoses:**

The patients were diagnosed with dental fluorosis of varying severity. Diagnosis was made based on clinical examination, history of fluoride intake, and radiographs. The condition was classified using the Thylstrup and Fejerskov index, which assesses the extent of fluorosis based on enamel opacity, pitting, and discoloration.

**Interventions:**

Case 1 (moderate fluorosis): The patient underwent home bleaching, composite restorations, and the application of laminate veneers after gingivectomy. Case 2 (moderate-to-severe fluorosis): The patient had several teeth extracted due to failed endodontic treatment, followed by the placement of lithium disilicate crowns and a zirconia bridge. Case 3 (mild fluorosis): This patient received in-office and at-home bleaching, followed by macro-abrasion and resin infiltration to improve the appearance of white spots.

**Outcomes:**

Each treatment successfully addressed the functional and aesthetic concerns of the patients. The use of bleaching, veneers, crowns, and resin infiltration significantly improved their smiles. All patients reported satisfaction with the results, achieving a more natural tooth appearance and improved confidence in their dental health.

**Lessons:**

This case series highlights the importance of a multi-modal, individualized approach to managing dental fluorosis. By tailoring treatment to the severity of the condition, aesthetic and functional improvements were consistently achieved across different fluorosis severities. Dental fluorosis management should prioritize minimally invasive options while maintaining a focus on long-term aesthetic and functional restoration.

## 1. Introduction

Dental fluorosis is a condition marked by enamel hypo-mineralization caused by high fluoride intake during tooth development. The condition’s severity varies, ranging from mild white opacities to severe brown pigmentation and enamel pitting that affect aesthetics and, in severe instances, dental function.^[1]^ Fluorosis not only affects the appearance of teeth but also increases the risk of caries and mechanical wear, particularly when enamel integrity is compromised. Dental fluorosis occurs due to ameloblast dysfunction, resulting in enamel with poor mineral content. Excessive fluoride intake during the critical stages of enamel formation increases the porosity of the enamel, resulting in distinct discolorations associated with fluorosis.^[2,3]^ There is a link between the intensity of the condition and the quantity of fluoride consumed, with more frequent exposures causing more severe enamel abnormalities.^[3]^

Fluorosis cases are prevalent in regions where drinking water naturally contains fluoride levels exceeding 1.5 mg/L, particularly in parts of Asia and Africa, where 51% and 46% of the population, respectively, are affected depending on the water’s fluoride concentration.^[4]^ Yemen, located in Asia south of Saudi Arabia, is ranked among the top ten countries most affected by high fluoride concentrations in groundwater, placing tenth globally, with an estimated 4.3 million people at risk of fluorosis.^[4]^ Furthermore, toothpaste and nutritional supplements may contribute to fluorosis in addition to water fluoridation when used as a public health measure.^[5]^ Dental fluorosis can cause light white streaks to severe brown stains and pits, with severity often determined by indices^[6]^ such as Dean’s Index^[7]^ or Thylstrup and the Fejerskov Index (TFI)^[8]^ (Table 1). Dental Fluorosis leads to significant aesthetic concerns, particularly with the anterior teeth, often resulting in a need for cosmetic dental treatment.

**Table 1:** Thylstrup and Fejerskov index (Fejerskov et al, 1988): mild (1–3), moderate (4–5), and severe (6–9).

TFI	Criteria
0	Enamel retains normal translucency even after prolonged air-drying.
1	Narrow white lines appear along the perikymata.
2	Smooth surface: Clear lines of opacity follow the perikymata, sometimes merging with adjacent lines. Occlusal surface: Scattered opaque areas <2 mm, with distinct opacity on cuspal ridges
3	Smooth surface: Irregular, cloudy opacities merge; visible perikymata between them. Occlusal surface: Large opaque areas. Worn sections appear normal but are bordered by opaque enamel.
4	Smooth surface: Entire surface shows significant opacity or looks chalky white. Less affected where attrition has occurred. Occlusal surface: Entirely opaque with pronounced attrition shortly after eruption.
5	Entire surface displays strong opacity with small pits (<2 mm in diameter) where the outermost enamel is lost.
6	Smooth surface: Horizontal bands of pits (<2 mm vertically). Occlusal surface: Enamel loss in confluent areas (<3 mm) and visible attrition.
7	Smooth surface: Irregular loss of outer enamel affecting less than half of the surface. Occlusal surface: Changes in morphology due to merging pits and significant attrition.
8	Loss of outer enamel covering more than half of the surface.
9	Major enamel loss alters surface anatomy. A cervical rim of intact enamel is often observed.

TFI = Thylstrup and Fejerskov index.

Dental fluorosis treatment aims to hide tooth discoloration, and a dentist can use cosmetic procedures to address it. The expense and success rates for fluorosis treatment vary considerably depending on the severity. Dental fluorosis compromises the aesthetics, confidence, and overall self-esteem of the patient as it impairs their social interaction. Hence, careful consideration is required to address these challenges and provide suitable treatment to the patient. Several options exist that can manage the stains and simultaneously transform them into a beautiful aesthetic appearance. Most treatment strategies aim to restore the integrity of the enamel and improve the patient’s appearance. Therefore, based on the extent of fluorosis and whether it involves superficial or deep stains, appropriate treatment is suggested.

Depending on severity, treatment options may vary:

### 1.1. Micro-abrasion/ macro-abrasion

Micro-abrasion is a procedure used to remove superficial discoloration or stains from the enamel of the teeth. It involves controlled removal of a thin layer of enamel using a mixture of abrasive particles (often pumice) and an acidic substance (such as hydrochloric acid). This technique is most used to treat mild to moderate enamel defects, such as white spots or brown stains caused by conditions like fluorosis

Macro-abrasion is a common treatment for mild to moderate cases of dental fluorosis. It involves the mechanical removal of a thin layer of enamel to minimize surface discoloration. When combined with polishing, this method can significantly improve the appearance of fluorosis-affected teeth. However, it may not eliminate deeper stains.

### 1.2. Bleaching

In cases where discoloration is the main concern, bleaching treatments, either in-office, at-home or both can be effective. Whitening agents like hydrogen peroxide or carbamide peroxide are used to lighten the affected teeth. However, bleaching is less effective in severe cases where the discoloration is deeply embedded in the enamel.

### 1.3. Veneers and crowns

For more severe cases of fluorosis, where enamel damage is extensive, cosmetic restorations such as veneers or crowns are often the preferred solution. Veneers, made of porcelain or composite resin, cover the front surface of the teeth, masking discoloration and restoring a natural appearance. Crowns are used when there is significant structural damage to the teeth, providing both aesthetic and functional restoration.

### 1.4. Composite bonding

Another option for moderate fluorosis is composite bonding. In this procedure, tooth-colored resin is applied to the surface of the teeth to cover discoloration and restore their appearance. Composite bonding can be more affordable than veneers and offers immediate results.

Managing dental fluorosis requires a tailored approach based on the severity of the condition and the patient’s aesthetic preferences. Mild cases may benefit from micro-abrasion/macro-abrasion and bleaching, while more severe cases often require cosmetic interventions like veneers or crowns. This case series focuses on 3 patients from the same family, each exhibiting different levels of dental fluorosis. Through a multi-modal treatment approach, the series demonstrates how individualized treatment plans can effectively restore both function and aesthetics, addressing the patients’ concerns while preserving as much natural tooth structure as possible. The goal of this report is to highlight the variety of treatment options available for dental fluorosis and emphasize the importance of tailoring interventions to each patient’s specific condition. This case series is unique as it addresses the multi-modal management of dental fluorosis within a single family. It provides the comprehensive treatment approach, unlike most studies that use just 1 treatment method. The treatment is customized based on the severity of each patient’s condition, ensuring better results. Treating 3 patients from the same family highlights how genetic and environmental factors might influence dental fluorosis. It also shows the need combining conservative and restorative methods to address both aesthetic and functional concerns in such cases. Using the TFI to assess the severity of dental fluorosis provides a clear and reliable way to plan treatment, making the approach easier for other clinicians to follow.

### 1.5. Patient satisfaction and follow-up

#### 1.5.1. Case series and objective

This case series demonstrates the effectiveness of a multi-modal treatment strategy for addressing dental fluorosis in 3 patients from the same family. A treatment plan tailored to the patient’s needs was established to rebuild aesthetics and function while maintaining as much natural tooth structure as possible. Written informed consent was obtained from the patients for case presentation and reporting. The ethical clearance was obtained from the institute ethical committee with ethical clearance number – IRB/KKUCOD/ETH/2024-25/009.

The first case demonstrates moderate fluorosis severity among all 3 cases, showing how bleaching followed by laminate veneers helped rebuild the smiles and confidence of the patients. The second case demonstrates a moderate-to-severe form of fluorosis with crowns and FPD to replace missing lower anterior teeth. The third case, exhibiting the mildest form of fluorosis among the 3, was treated first with in-office and home bleaching, followed by macro-abrasion and resin infiltration. This series additionally highlights the importance of treating dental fluorosis holistically and individually, especially when multiple family members are affected.

#### 1.5.2. Case 1

A 24-year-old single Yemeni male patient visited the restorative department in College of Dentistry, King Khalid University, Abha, Saudi Arabia, with the complaint of discolored teeth and a desire to improve his smile. He grew up in a region with an increased amount of fluoride in the water distribution channel. He also had a history of similar discoloration in primary teeth, and the condition runs in his family. The patient had no abnormal findings in his medical history and had no known contraindications for dental procedures.

#### 1.5.3. Intraoral examination

**Figure 1.**
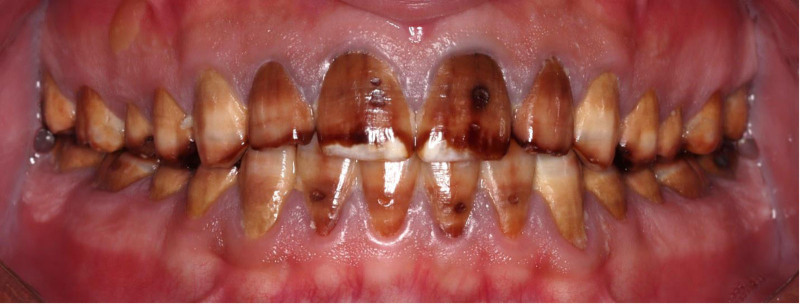
Preoperative frontal view.

**Figure 2.**
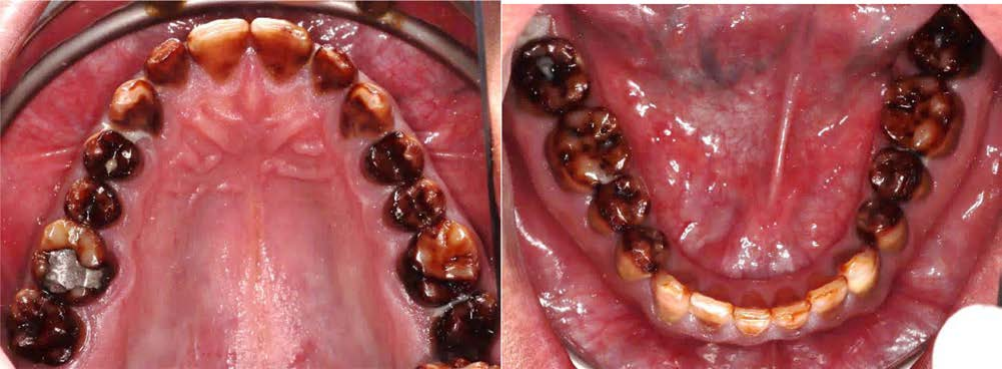
Preoperative occlusal view.

**Figure 3.**
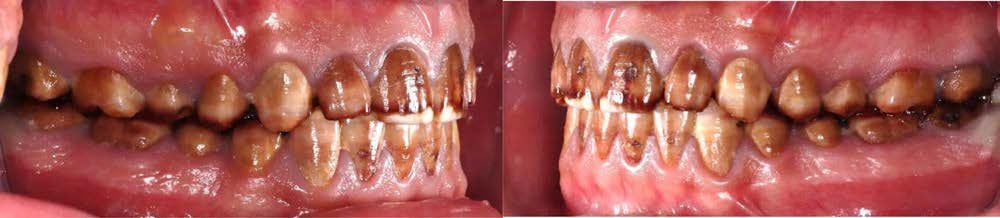
Preoperative right and left lateral view.

The patient had fair oral hygiene, with mild plaque present. Stippled gingiva was observed with mild generalized marginal redness and edema. There was no loss of clinical attachment level, nor was any bleeding on probing observed. Dental fluorosis was noticed on almost all teeth (TFI 4–5), the entire surface exhibits marked opacity with brown stain on every surface, in addition to pitted surfaces (Figs. 1–3). The only restoration present was in 1 posterior tooth, #16. Multiple carious lesions were noted, including in teeth #17, 15, 14, 11, 21, 37, 36, 35, 31, 32, 33, 41, 42, 43, 45, 46, and 47.

**Figure 4.**
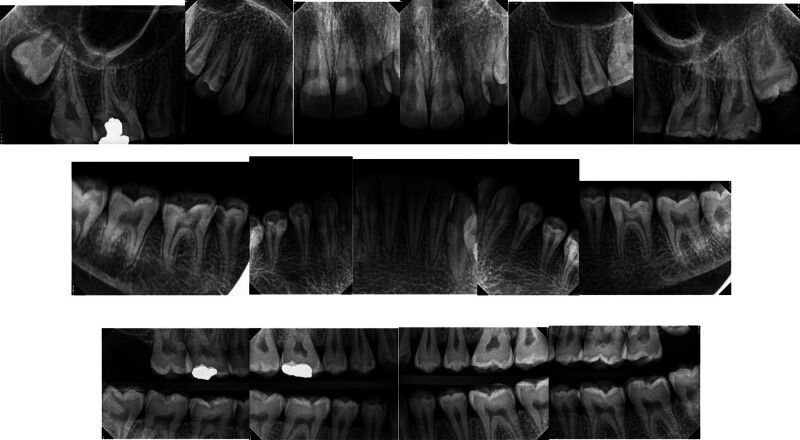
Preoperative full-mouth x-rays.

Angle Class I molar and canine occlusion were observed. A series of full-mouth radiographs were obtained, identifying healthy endodontic and periodontal conditions (Fig. 4). As previously mentioned, several posterior teeth showed interproximal carious lesions.

#### 1.5.4. Treatment rendered

**Figure 5.**
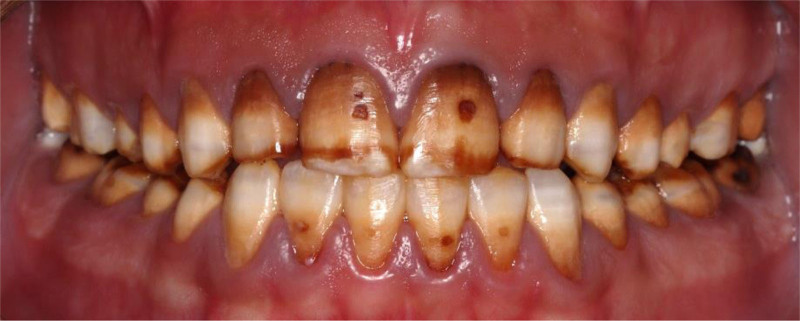
Post home bleach treatment.

Following the esthetic and tooth analysis, a treatment plan was developed. During Phase I, preventive treatment included supragingival scaling and oral hygiene instructions. Phase II: Operative treatment included home bleaching with 20% carbamide peroxide (Opalescence, Ultradent, South Jordan) for 7 to 10 days (Fig. 5), followed by composite Class I restorations for teeth 17, 15, 14, 37, and 47, and Class II restorations for teeth 45 and 46. Isolation with a thin/medium-thickness rubber dam was used and proximal buildup were achieved using a sectional matrix Palodent (Dentsply Sirona, Charlotte). A 37% phosphoric acid Scotchbond Universal Etchant (3M, Maplewood) was used for etching, with the total etch technique. Direct composite restoration was completed using a universal bonding agent and IPS Empress (Ivoclar Vivadent, Schaan, Liechtenstein). Diacomp Twist (EVE Ernst Vetter GmbH, Pforzheim, Germany) and Sof-Lex strips (3M) were used for polishing.

**Figure 6.**
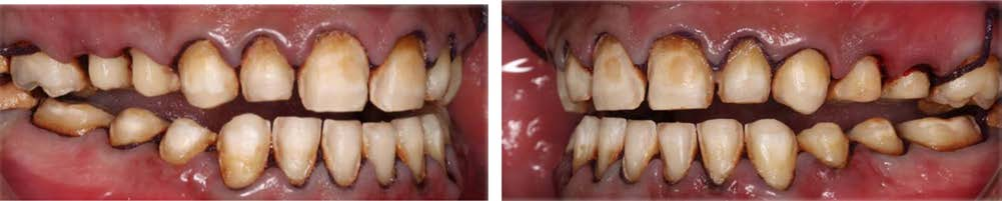
Veneers tooth preparation.

Phase IV: Pre-prosthetic/surgical treatment included a diagnostic wax-up and a 1 to 1.5 mm gingivectomy using electrosurgery on teeth 14, 15, 24, and 25 to increase the length of the clinical crown for better esthetic. Phase V: The prosthetic phase began after 12 weeks of tissue healing when the teeth were ready for laminate veneers. The butt joint preparation design was used, standard procedures were followed to finish the teeth preparations16 to 26 and 36 to 46 for veneers (Fig. 6). To facilitate impression-making, retraction cord (knitted) sizes 0 and 00 Ultrapak (Ultradent), as well as a hemostatic agent ViscoStat (Ultradent), were used. The definitive pressed lithium disilicate veneers were developed to match the occlusal and contour of the provisional restorations after final impressions were taken using Penta H polyvinyl siloxane (heavy and light body) (3M ESPE, Seefeld, Germany). Shade selection was done using the Vitapan Classical shade guide (VITA Zahnfabrik, Bad Säckingen, Germany). Protemp was used for temporization purposes (3M). Before cementation, the teeth surfaces were first acid etched with 37% phosphoric acid (Ultra-Etch, Ultradent) for a period of 15 seconds and a universal adhesive system Peak Universal Bond (Ultradent) was applied. The lithium disilicate veneers were conditioned by hydrofluoric acid application 9% Porcelain Etch for 20 seconds (Ultradent) and silane application for 60 seconds (Monobond Plus, Ivoclar Vivadent) to prepare them for optimal bonding. The intaglio surface of each restoration was coated with a light-cured resin cement (Variolink Esthetic LC, Ivoclar Vivadent) and spot-cured for 2 seconds per tooth after the restorations were fully seated on the prepared tooth surface. Excess cement was removed, and all accessible surfaces were fully light cured for 20 seconds. Excess cement was removed using scalers, and the occlusion was adjusted to produce even bilateral contacts and an occlusal scheme that was mutually protected.

**Figure 7.**
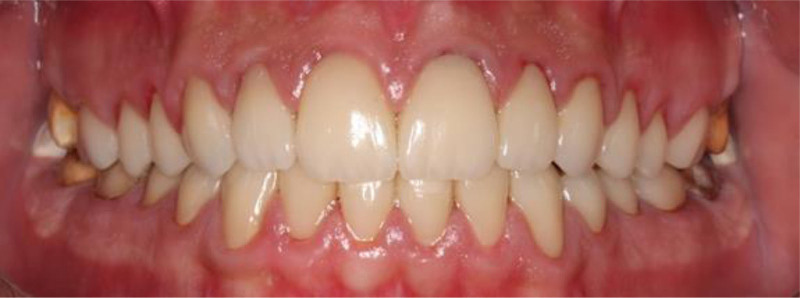
Frontal view with final restoration.

**Figure 8.**
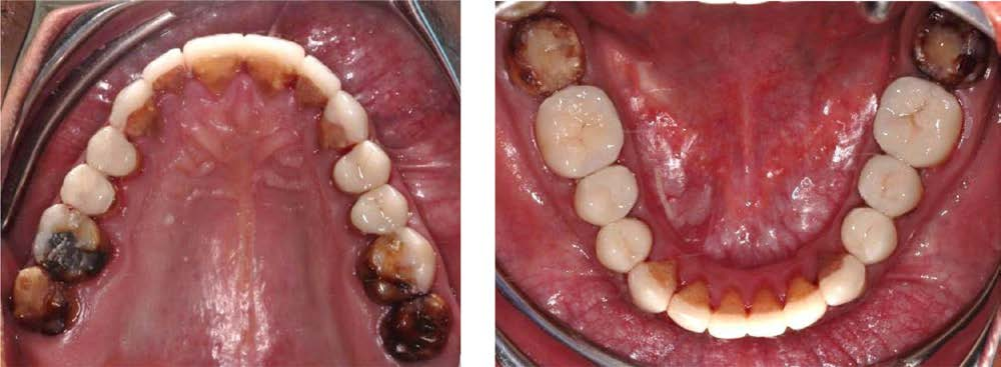
Occlusal view with final restorations.

**Figure 9.**
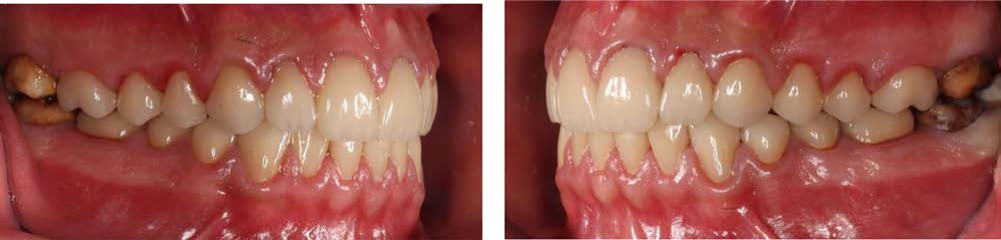
Right and left lateral view with final restorations.

Posttreatment, the patient’s dental esthetics and function improved significantly (Figs. 7–9). The patient expressed satisfaction with the aesthetic outcomes, including complete masking of the fluorotic structures and a smile line that blended with the facial structures. A 3-month recall schedule was given to the patient to support healthy periodontal and dental conditions.

#### 1.5.5. Case 2

A 25-year-old married Yemeni male patient visited the prosthodontic department in College of Dentistry, King Khalid University, Abha, Saudi Arabia, with a complaint like that of Case 1: yellow/brown teeth and a desire to improve his smile. He grew up in a region with an increased amount of fluoride content in the well water. He reported a history of discoloration of permanent teeth only, which worsened as he aged. The patient had no abnormal findings in his medical history and no known contraindications for dental procedures.

#### 1.5.6. Intraoral examination

**Figure 10.**
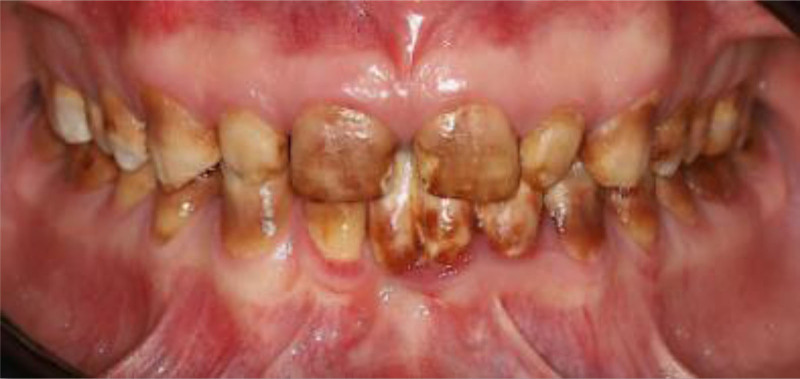
preoperative frontal view.

**Figure 11.**
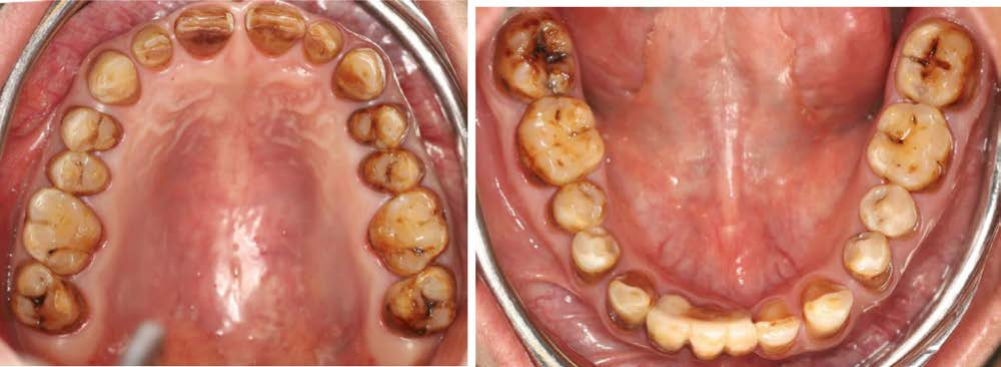
Preoperative occlusal view.

**Figure 12.**
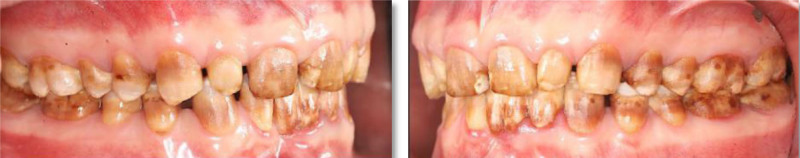
preoperative right and left lateral view.

**Figure 13.**
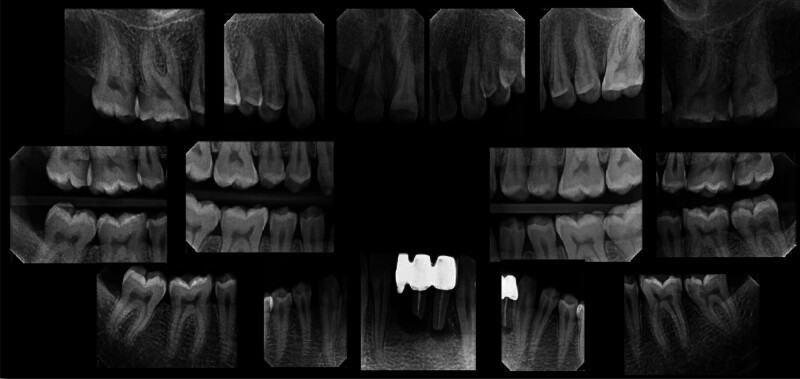
Preoperative full-mouth X-rays.

The patient had fair oral hygiene with generalized gingivitis. Probing depths ranged between 3 and 4 mm, with no bleeding on probing noted. Dental fluorosis was observed in almost all teeth (TFI 5–6), characterized by marked opacity and brown stains with focal loss of outermost enamel. Furthermore, wear from habitual tooth grinding exacerbated the condition, making the patient a candidate for comprehensive restorative care (Figs. 10–12). Intraorally, A cantilever bridge, using teeth #31 and #32 as abutments to replace the missing tooth #41 was there. Multiple carious lesions were noted on teeth 16, 17, 26, 27, 36, 37, and 47. Angle Class I molar and canine occlusion were observed. A series of full-mouth radiographs were obtained, identifying healthy endodontic and periodontal conditions (Fig. 13). Considering the missing tooth 41 and failed endodontically treated teeth 31 and 32, 2 treatment plan options were proposed: 1 involving implant placement and the other a 6-unit bridge. The patient opted for the bridge instead of the implant due to cost and time concerns.

#### 1.5.7. Treatment rendered

**Figure 14.**
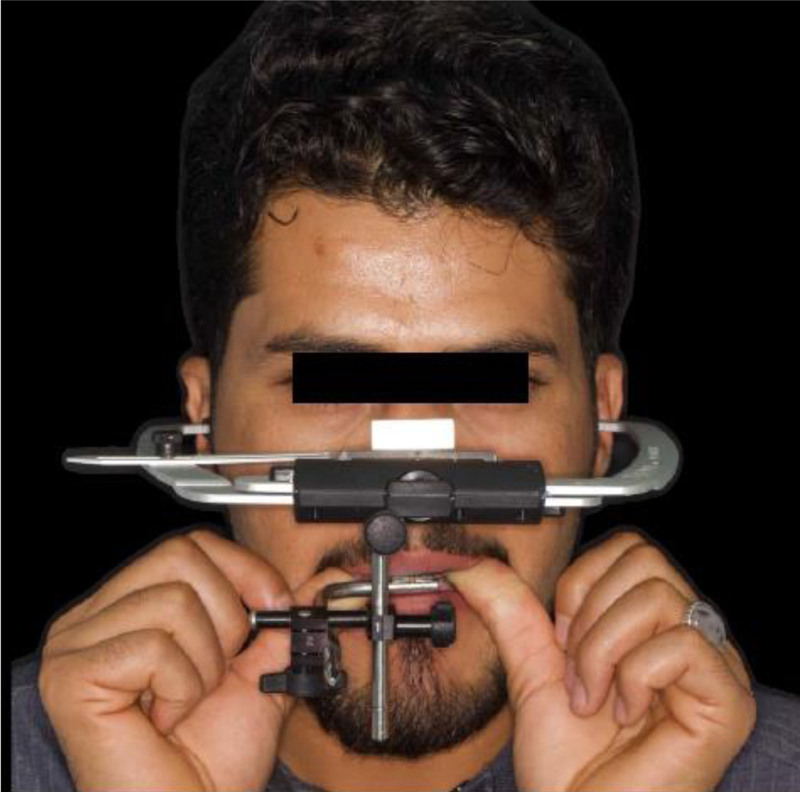
Face-bow transfer.

**Figure 15.**
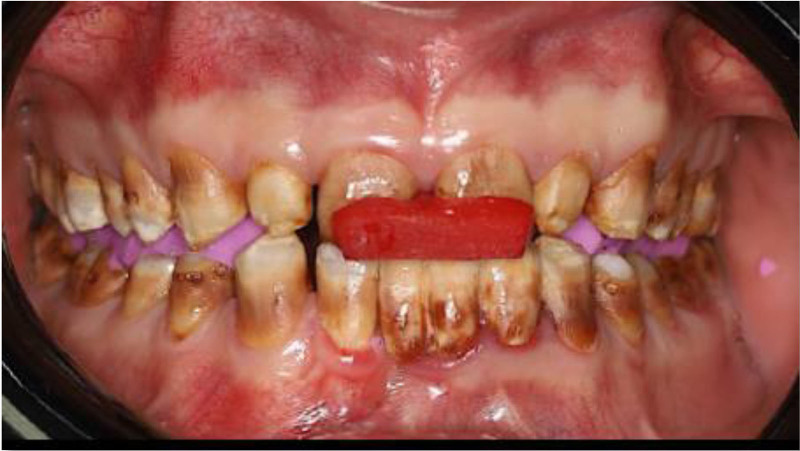
Centric relation records.

**Figure 16.**
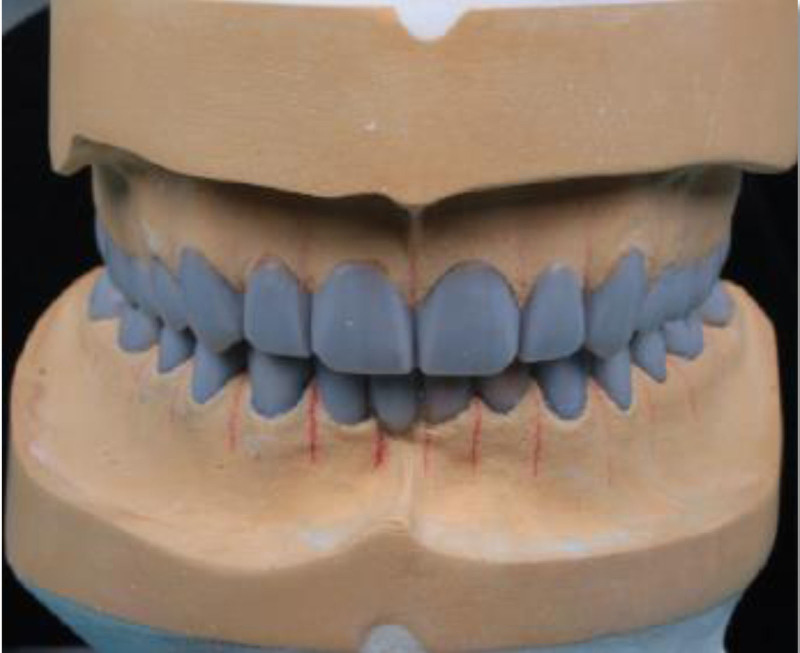
Diagnostic wax-up.

**Figure 17.**
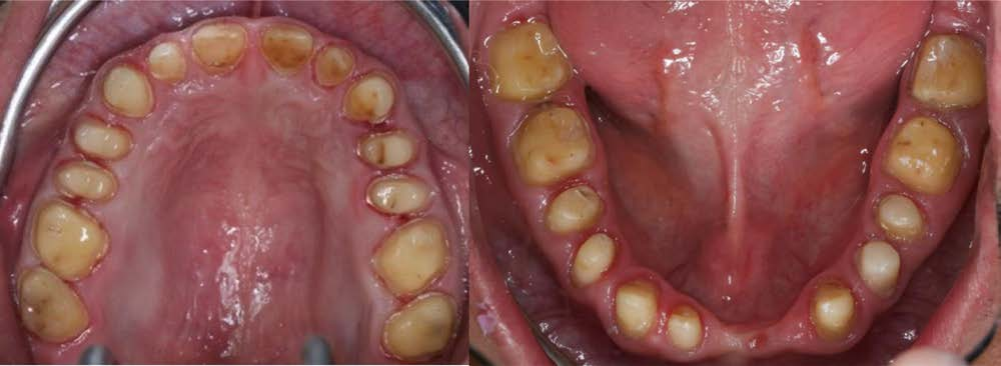
Teeth preparation.

**Figure 18.**
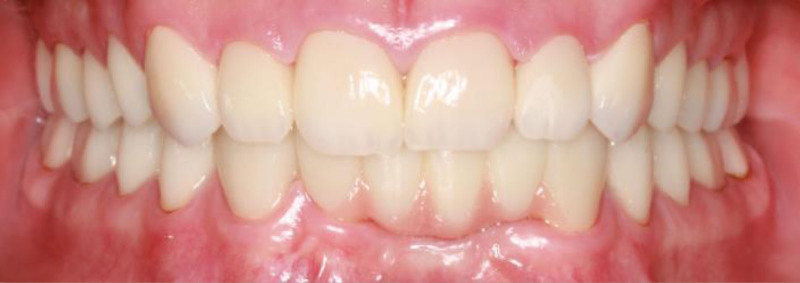
Frontal view with final restorations.

**Figure 19.**
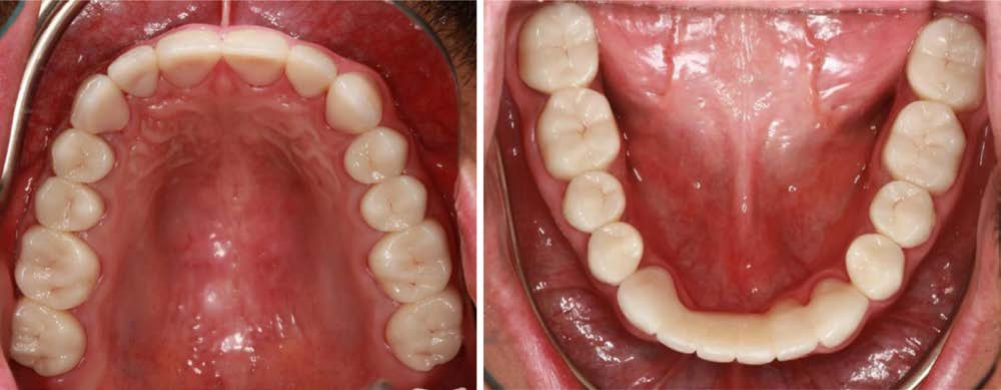
Occlusal view with final restorations.

**Figure 20.**
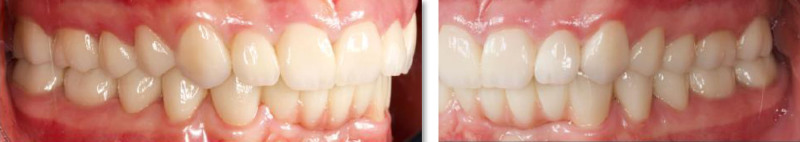
Right and left lateral views with final restorations.

**Figure 21.**
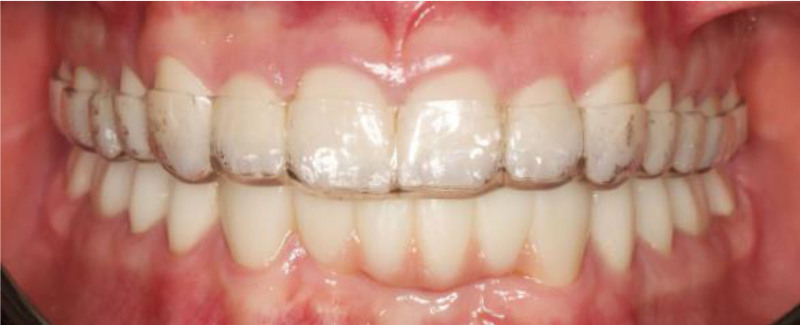
Occlusal appliance.

Following the esthetic and tooth analysis, a treatment plan was developed. During Phase I, preventive treatment included supragingival scaling and oral hygiene instructions. Phase II, the surgical phase, included the extraction of teeth 31 and 32 due to failed endodontic treatments and extensive structural damage. The teeth were non-restorable. Phase III, prosthetic treatment, included face-bow transfer (Fig. 14) and recording of centric relation (Fig. 15), followed by diagnostic wax-up (Fig. 16). Once the patient was satisfied, tooth preparation began after the healing of the extracted sockets. Standard procedures were followed to complete the crown preparations and achieve the necessary reduction in material thickness for all teeth (Fig. 17). Impression-making and shade selection were carried out following the same protocol as in Case 1, utilizing Penta H polyvinyl siloxane (3M ESPE, Seefeld, Germany) for the impressions and the Vitapan Classical shade guide (VITA Zahnfabrik, Bad Säckingen, Germany) for shade selection. An interocclusal record was taken using a bite registration material Regisil PB (Dentsply Sirona, Charlotte) to accurately register the occlusion and ensure proper alignment of the restorations. Lithium disilicate crowns were prepared for all teeth except for the FPD, which was replaced with layered zirconia for the lower anterior teeth # 33 to 43 replacing the missing 41,31,32 teeth. Temporization was achieved using Protemp (3M) for the crowns, ensuring patient comfort and stability during the interim phase. For final cementation of the crowns, self-adhesive resin cement Relyx (3M) was used. Before cementation, The Lithium disilicate crowns were conditioned by 9% hydrofluoric acid application for 20 seconds (Porcelain Etch, Ultradent) and silane application for 60 seconds Monobond Plus (Ivoclar Vivadent) to prepare them for optimal bonding. The crowns were loaded with cement, seated, and initially cured for 2 seconds. After removing the excess cement, each surface was cured for 20 seconds. For the Zirconia FPD, sandblasting with 50 µm aluminum oxide was performed before loading with cement and seating. Occlusal adjustments were then made. (Figs. 18–20) Following delivery, the patient was provided with a hard acrylic maxillary occlusal guard (Fig. 21) to protect the crowns and the FPD from the habitual tooth grinding that the patient is having. A 3-month recall schedule was given to the patient to support healthy periodontal and dental conditions.

#### 1.5.8. Case 3

A 23-year-old Yemeni male patient presented to the restorative department in the College of Dentistry, King Khalid University, Abha, Saudi Arabia. Like the previous patients, he had consumed fluoride-rich water. He sought treatment to change the color of his teeth. He also had a history of primary tooth discoloration, which runs in his family. His medical history was unremarkable, with no contraindications to dental procedures. However, he has a habit of consuming khat.

#### 1.5.9. Intraoral examination

**Figure 22.**
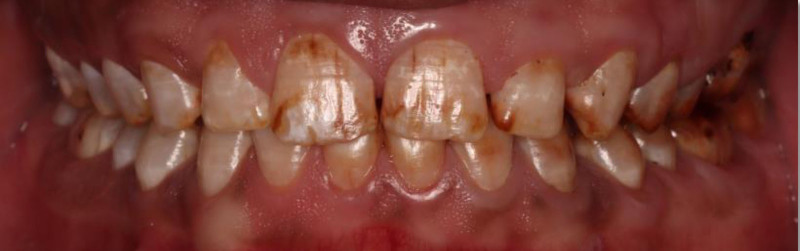
Preoperative frontal view.

**Figure 23.**
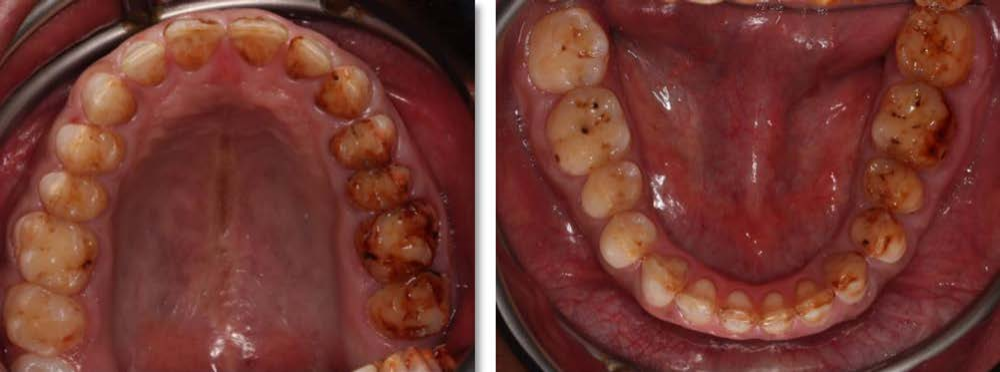
Preoperative occlusal view.

**Figure 24.**
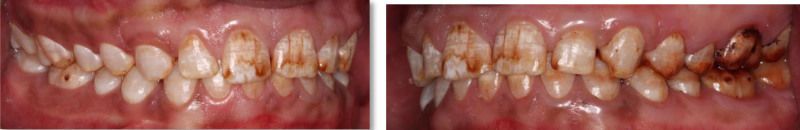
Preoperative right and left lateral view.

**Figure 25.**
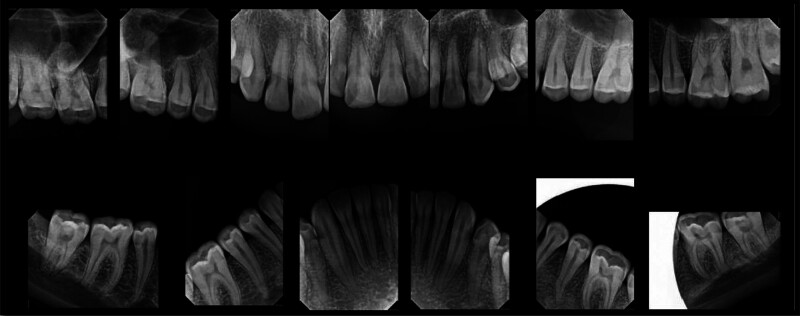
Preoperative Full-mouth x-rays.

The patient exhibited generalized marginal gingivitis. Probing depth ranged around 3 mm, with no bleeding on probing noted. Most of the maxillary and mandibular dentition showed enamel fluorosis (TFI 3–4), characterized by paper-white opacities on every surface and brown stains (Figs. 22–24). Intraorally, multiple caries was noted, including teeth 16, 23, 26, 36, and 46, along with dental fluorosis. Angle Class I molar and canine occlusion were observed. A series of full-mouth radiographs were obtained, which identified healthy endodontic and periodontal conditions (Fig. 25).

#### 1.5.10. Treatment rendered

**Figure 26.**
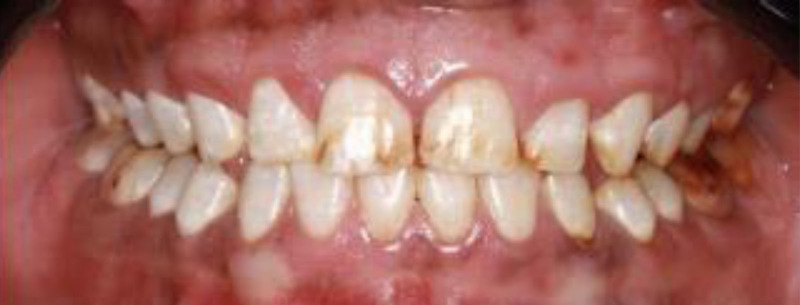
Post in-office and home bleaching.

**Figure 27.**
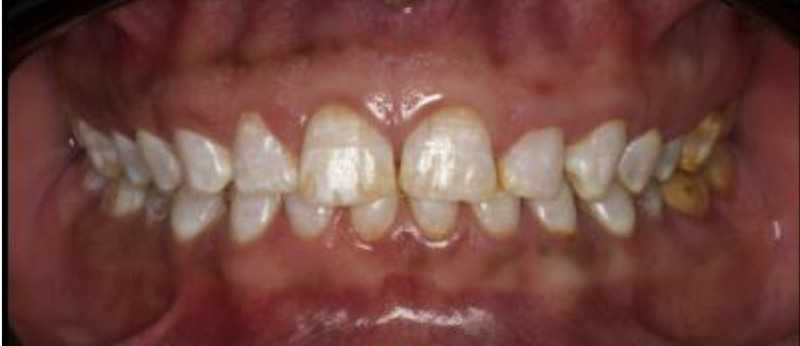
Post macro-abrasion.

Following the esthetic and tooth analysis, a treatment plan was created. During Phase I, preventive treatment consisted of supragingival scaling and oral hygiene instructions. Phase II operative treatment included the restoration of teeth 16, 23, 26, 36, and 46. After that, in-office bleaching was performed using a 35% hydrogen peroxide gel (Opalescence Boost, Ultradent), and home bleaching was recommended for 7 to 10 days using 20% carbamide peroxide (Opalescence, Ultradent) (Fig. 26). After the bleaching treatment is over, macro-abrasion was done to improve the cosmetic appearance of teeth with mild fluorosis. The external or unsightly stained layer of enamel that harbored the irregularity was removed using rotary instruments (fine diamond bur). Special care was taken during the macro-abrasion procedure, so the enamel loss was kept to a minimum while attaining smoother surface of the teeth. After the macro-abrasion, rubber cups and a prophylactic paste were employed to restore the teeth luster. The teeth shade changed dramatically after the bleaching and macro-abrasion (Fig. 27).

**Figure 28.**
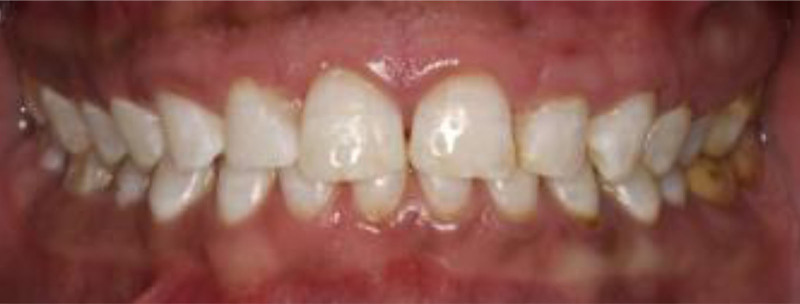
Post resin infiltration frontal view.

**Figure 29.**
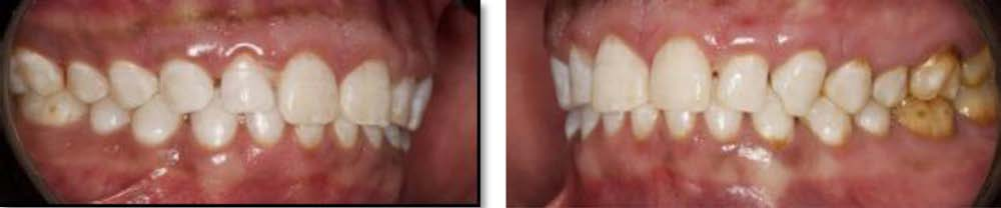
Post resin Infiltration right and left lateral views.

Furthermore, resin infiltration (Icon-Etch, DMG America, Englewood) was used To Improve the appearance of the white spots 2 weeks after the bleaching. A rubber dam was used to create isolation, and the teeth’s surface was etched with 15% hydrochloric acid for 120 seconds, rinsed with water for 30 seconds, and allowed to air dry. Then, 99% ethanol was applied for 30 seconds, and after drying, the resin was left in place for 3 minutes. Any excess material was removed, dental floss was used to clear interproximal spillage, and the area was photopolymerized for 40 seconds. A second infiltration was used, it was left for 1 minute before being polymerized for 40 seconds. The surface was then polished and finished with abrasive strips (Sof-Lex, 3M). The patient was extremely satisfied and happy with the outcome (Figs. 28 and 29). Like previous cases, follow-up appointments were scheduled every 3 months.

## 2. Discussion

Treatment options for dental fluorosis include tooth whitening, micro-abrasion, and prosthetic rehabilitation with fixed restorations.^[9]^ The severity of the condition and the individual’s aesthetic choices guide treatment decisions. It is best to try more conservative modalities in mild to moderate cases, particularly given that as the patient approaches young adulthood, their appearance may progressively improve.^[10]^ In severe dental fluorosis cases, there is a tendency for the enamel to chip and break, causing noticeable yellowing and structural flaws that require the incorporation of indirect restorations. A person’s teeth can experience a variety of consequences from fluorosis.

In Case 1, almost all teeth were affected by moderate fluorosis. To address the problems and enable a less invasive way to restore esthetics of dentitions laminate veneers were determined to be required to meet our treatment objectives since there was some enamel loss and the discoloration was not totally improved with the bleaching alone. In addition, both the upper first and second premolars underwent pre-prosthetic gingivectomy to increase the length of the clinical crown facially for better esthetics. Lithium disilicate veneers was selected as the restoration material for this case based on its proven clinical effectiveness,^[11,12]^ its suitability for bonding to enamel and dentin,^[13]^ excellent esthetics, and minimally invasive preparation requirements. Bleaching was used to lighten the discoloration. According to recent studies, adhesively bonded posterior lithium disilicate partial coverage restorations can be successfully placed at depths of <1 mm^[14–16]^ allowing conservative occlusal reduction for veneers when needed. Our cautious approach avoided overly whenever possible to avoid exposing the pulp or causing hypersensitivity by preserving the occlusal tooth structure. Despite the compromised bonding surfaces of fluorosed enamel, in vitro studies have demonstrated that incorporating phosphoric acid etching improves bond strength to enamel in both moderately and severely fluorosed teeth.^[17]^

In Case 2, the condition presented with moderate-to severe discoloration affecting most teeth, along with signs of worn tooth surfaces from habitual behaviors such as tooth grinding. In conjunction with the fluorosis, this wear guided the decision for prosthetic intervention using lithium disilicate crowns and layered zirconia bridge to replace the missing lower anterior teeth. This case was restored using lithium disilicate crowns for all teeth except the lower anterior region which was replaced with layered zirconia to replace the missing teeth.

In Case 3, both bleaching and abrasion techniques were applied to the patient due to their mild to moderate fluorosis. In most cases, patients with a golden teeth appearance due to fluorosis require a combined therapy regimen to achieve the desired aesthetic outcome.

Both at-home and in-office bleaching were employed, along with macro-abrasion. This procedure’s main advantage is that it can be finished with the least chairside time and is not as invasive as other restorative procedures. To get the desired outcome and improve the appearance of the white spots, resin infiltration was done after this. The most efficient, quick, and promising method is the resin infiltration technique.^[18]^ This satisfies the need for minimally invasive cosmetic dentistry and avoids needless tissue removal while producing satisfactory results. Following the etching of the enamel’s surface for 120 seconds with a 15% hydrochloric acid solution, the resin penetrates the fluorosis lesion. By removing 58 µm (37 µm), the most outer layer of enamel, the hypo-mineralized site is made visible. So, the porous enamel is traversed by a low viscosity resin.^[19,20]^

Differentiating dental fluorosis from Molar Incisor Hypo-mineralization (MIH) and Amelogenesis Imperfecta is crucial in the diagnostic process. The most critical information for differentiating dental fluorosis from other conditions includes the patient’s family history, the location of residence, and the time span over which the discoloration first appeared.^[3,9]^ Despite these findings, dental fluorosis remains challenging to distinguish histologically and clinically from other forms of hypo-mineralized and hypoplastic enamel.^[21]^

Across all 3 cases, it is evident that a multi-modal treatment approach can be highly effective in managing the various clinical presentations of dental fluorosis. Conservative methods, such as bleaching and micro/macro-abrasion, are appropriate for mild to moderate cases, whereas more extensive restorative techniques, including veneers and crowns, are necessary for severe fluorosis. This progression underscores the need for individualized treatment plans that consider both the extent of enamel damage and the patient’s aesthetic desires.

In addition to aesthetic restoration, the functional impact of fluorosis must be considered. Enamel compromised by fluorosis is more prone to caries, mechanical wear, and potential fracture. This was particularly evident in Case 2, where the patient’s enamel attrition and tooth grinding necessitated a protective occlusal guard posttreatment. For such patients, long-term management strategies should include both restorative care and preventive measures, such as the use of occlusal appliances to prevent further wear.

Moreover, patient education is a key component in managing dental fluorosis. Patients need to be informed about the nature of their condition, the causes of fluorosis, and the range of treatment options available. In areas where fluoride exposure is high, public health initiatives should also emphasize the importance of monitoring fluoride intake to prevent fluorosis, especially in children during the critical stages of tooth development.

This paper aims to provide an overview of the various dental fluorosis treatment options, ranging from extensive laminate veneers, FPD, and crowns to more conservative treatments like bleaching, macro-abrasion, and resin infiltration. Therefore, it is essential to ensure that the dentist is informed about every possible treatment option for the benefit of both the patient and the dentist.

## 3. Conclusion

This case series demonstrates that the management of dental fluorosis requires a flexible, individualized approach, with treatment options tailored to the severity of the condition and the patient’s specific needs. Dental fluorosis can manifest in various ways, varying in severity and extent, even within an individual. Therefore, a proper course of treatment must be chosen after a careful assessment of the affected teeth and the individual’s overall functional and aesthetic requirements. In this series of cases, consistent and favorable treatment outcomes were achieved using bleaching, resin infiltration, adhesively bonded lithium disilicate restorations, and macro-abrasion. To successfully address the functional and esthetic aspects of severely fluorosed dentition, the dentist must be informed about all available options. By addressing both aesthetic and functional concerns, comprehensive treatment strategies can successfully restore the patient’s confidence and oral health.

## Author contributions

**Conceptualization:** Ghadeer Saleh Alwadai, Salha Hussein Huaylah, Mashael Khalid Althobati, Fahad Amer AlMutairi.

**Data curation:** Ghadeer Saleh Alwadai, Salha Hussein Huaylah, Mashael Khalid Althobati, Fahad Amer AlMutairi.

**Formal analysis:** Ghadeer Saleh Alwadai, Salha Hussein Huaylah, Mashael Khalid Althobati, Fahad Amer AlMutairi.

**Funding acquisition:** Ghadeer Saleh Alwadai, Salha Hussein Huaylah, Mashael Khalid Althobati, Fahad Amer AlMutairi.

**Investigation:** Ghadeer Saleh Alwadai, Salha Hussein Huaylah, Mashael Khalid Althobati, Fahad Amer AlMutairi.

**Methodology:** Ghadeer Saleh Alwadai, Salha Hussein Huaylah, Mashael Khalid Althobati, Fahad Amer AlMutairi.

**Project administration:** Ghadeer Saleh Alwadai, Salha Hussein Huaylah, Mashael Khalid Althobati, Fahad Amer AlMutairi.

**Resources:** Ghadeer Saleh Alwadai, Salha Hussein Huaylah, Mashael Khalid Althobati, Fahad Amer AlMutairi.

**Software:** Ghadeer Saleh Alwadai, Salha Hussein Huaylah, Mashael Khalid Althobati, Fahad Amer AlMutairi.

**Supervision:** Ghadeer Saleh Alwadai, Salha Hussein Huaylah, Mashael Khalid Althobati, Fahad Amer AlMutairi.

**Validation:** Ghadeer Saleh Alwadai, Salha Hussein Huaylah, Mashael Khalid Althobati, Fahad Amer AlMutairi.

**Visualization:** Ghadeer Saleh Alwadai, Salha Hussein Huaylah, Mashael Khalid Althobati, Fahad Amer AlMutairi.

**Writing – original draft:** Ghadeer Saleh Alwadai, Salha Hussein Huaylah, Mashael Khalid Althobati, Fahad Amer AlMutairi.

**Writing – review & editing:** Ghadeer Saleh Alwadai, Salha Hussein Huaylah, Mashael Khalid Althobati, Fahad Amer AlMutairi.

## References

[1] Lee JD Inoue N Lee C Park S Lee SJ. Comprehensive management of severe dental fluorosis with adhesively bonded all-ceramic restorations. Prosthesis. 2021;3:194–208.

[2] Das G Tirth V Arora S, . Effect of fluoride concentration in drinking water on dental fluorosis in Southwest Saudi Arabia. Int J Environ Res Public Health. 2020;17:3914. 32492867 10.3390/ijerph17113914 PMC7312808

[3] Fejerskov O Larsen MJ Richards A Baelum V. Dental tissue effects of fluoride. Adv Dent Res. 1994;8:15–31. 7993557 10.1177/08959374940080010601

[4] Podgorski J Berg M. Global analysis and prediction of fluoride in groundwater. Nat Commun. 2022;13:4232. 35915064 10.1038/s41467-022-31940-x PMC9343638

[5] Ismail AI Hasson H. Fluoride supplements, dental caries and fluorosis: a systematic review. J Am Dent Assoc. 2008;139:1457–68. 18978383 10.14219/jada.archive.2008.0071

[6] Khyalia P Jugiani H Dangi J Laura JS Nandal M. A comprehensive review on the techniques and indexes used for the analysis of fluorosis in humans and cattle. Orient J Chem. 2023;39.

[7] Dean HT. Classification of mottled enamel diagnosis. J Am Dent Assoc (1922). 1934;21:1421–6.

[8] Thylstrup A Fejerskov O. Clinical appearance of dental fluorosis in permanent teeth in relation to histologic changes. Community Dent Oral Epidemiol. 1978;6:315–28. 282114 10.1111/j.1600-0528.1978.tb01173.x

[9] Akpata ES. Occurrence and management of dental fluorosis. Int Dent J. 2001;51:325–33. 11697585 10.1002/j.1875-595x.2001.tb00845.x

[10] Curtis AM Levy SM Cavanaugh JE Warren JJ Kolker JL Weber-Gasparoni K. Decline in dental fluorosis severity during adolescence: a cohort study. J Dent Res. 2020;99:388–94. 32091961 10.1177/0022034520906089 PMC7088205

[11] Malament KA Natto ZS Thompson V Rekow D Eckert S Weber HP. Ten-year survival of pressed, acid-etched e.max lithium disilicate monolithic and bilayered complete-coverage restorations: performance and outcomes as a function of tooth position and age. J Prosthet Dent. 2019;121:782–90. 30955942 10.1016/j.prosdent.2018.11.024

[12] Pieger S Salman A Bidra AS. Clinical outcomes of lithium disilicate single crowns and partial fixed dental prostheses: a systematic review. J Prosthet Dent. 2014;112:22–30. 24674802 10.1016/j.prosdent.2014.01.005

[13] Rojpaibool T Leevailoj C. Fracture resistance of lithium disilicate ceramics bonded to enamel or dentin using different resin cement types and film thicknesses. J Prosthodont. 2017;26:141–9. 26505488 10.1111/jopr.12372

[14] Luciano M Francesca Z Michela S Tommaso M Massimo A. Lithium disilicate posterior overlays: clinical and biomechanical features. Clin Oral Investig. 2020;24:841–8. 10.1007/s00784-019-02972-3 PMC13161036 31201516

[15] Sasse M Krummel A Klosa K Kern M. Influence of restoration thickness and dental bonding surface on the fracture resistance of full-coverage occlusal veneers made from lithium disilicate ceramic. Dent Mater. 2015;31:907–15. 26051232 10.1016/j.dental.2015.04.017

[16] Baldissara P Monaco C Onofri E Fonseca RG Ciocca L. Fatigue resistance of monolithic lithium disilicate occlusal veneers: a pilot study. Odontology. 2019;107:482–90. 30840218 10.1007/s10266-019-00417-7

[17] Weerasinghe DS Nikaido T Wettasinghe KA Abayakoon JB Tagami J. Micro-shear bond strength and morphological analysis of a self-etching primer adhesive system to fluorosed enamel. J Dent. 2005;33:419–26. 15833398 10.1016/j.jdent.2004.11.004

[18] Di Giovanni T Eliades T Papageorgiou SN. Interventions for dental fluorosis: a systematic review. J Esthet Restor Dent. 2018;30:502–8. 30194793 10.1111/jerd.12408

[19] Mazur M Westland S Guerra F. Objective and subjective aesthetic performance of icon^®^ treatment for enamel hypomineralization lesions in young adolescents: a retrospective single center study. J Dent. 2018;68:104–8. 29104142 10.1016/j.jdent.2017.11.001

[20] Zotti F Albertini L Tomizioli N Capocasale G Albanese M. Resin infiltration in dental fluorosis treatment-1-year follow-up. Medicina (Kaunas). 2020;57:22. 33383755 10.3390/medicina57010022 PMC7823358

[21] Sabokseir A Golkari A Sheiham A. Distinguishing between enamel fluorosis and other enamel defects in permanent teeth of children. PeerJ. 2016;4:e1745. 26966672 10.7717/peerj.1745 PMC4782718

